# Cycloserine resistance among drug-resistant tuberculosis cases in Taiwan

**DOI:** 10.1128/spectrum.03422-24

**Published:** 2025-06-09

**Authors:** Sheng-Han Wu, Hsin-Yi Liu, Kuang-Hung Liu, Hseuh-Chien Hsiao, Ruwen Jou

**Affiliations:** 1 Tuberculosis Research Center, Centers for Disease Control, Ministry of Health and Welfare, Taipei, Taiwan; 2 Reference Laboratory of Mycobacteriology, Centers for Disease Control, Ministry of Health and Welfare, Taipei, Taiwan; MultiCare Health System, Tacoma, Washington, USA

**Keywords:** tuberculosis, *Mycobacterium tuberculosis*, cycloserine, drug susceptibility testing, critical concentration, drug resistance

## Abstract

Cycloserine (CS) is a widely used drug for drug-resistant tuberculosis (DR-TB) treatment. The World Health Organization (WHO) recently suggested a critical concentration (CC) for CS using the MGIT 960 system (MGIT). To strengthen our DR-TB management program, we performed CS resistance analyses to provide comprehensive drug susceptibility testing (DST). This retrospective study included *Mycobacterium tuberculosis* complex (MTBC) isolates obtained from 114 rifampin-resistant (RR) and multidrug-resistant (MDR) TB cases. We compared the results of phenotypic DST (pDST) and genotypic DST (gDST) and evaluated the minimum inhibitory concentration (MIC) using both MGIT and the Sensititre MYCOTB MIC Plate (Sensititre). Sanger sequencing and whole-genome sequencing were conducted to analyze the mutations of CS-resistant-associated *ald* and *alr* genes. Our results indicated that the optimal consistency with gDST was achieved with the CC of 16 µg/mL for MGIT, which aligns with the WHO recommendations, and the CC of 8 µg/mL for Sensititre. Of the 114 MTBC isolates, we found 5 (4.4%) CS-MGIT-resistant isolates, which all harbored mutations in the *alr* gene, including three previously known mutations M343T, T20M, L113R, and a novel mutation R243S, whereas seven low-MIC isolates harboring *alr* Q30R mutations might not be associated with CS resistance. Notably, M1I, E118K, and A184T in the *ald* gene and L113R, R243S, S261N, and M343T in the *alr* gene were predicted to have a destabilizing effect, which could interfere with protein functions and induce drug resistance. For accurate routine diagnosis of CS susceptibility, we adopted the CC of 16 µg/mL and suggested an interim 8 µg/mL using MGIT and Sensititre, respectively.

**IMPORTANCE:**

To strengthen the DR-TB management program in Taiwan, we performed cycloserine (CS) resistance analyses to enhance treatment outcomes. Of the 114 drug-resistant tuberculosis (DR-TB) isolates, we found 5 (4.4%) CS-MGIT-resistant isolates, with four isolates classified as multidrug-resistant (MDR)-TB and one isolate as Pre-XDR-TB. In addition, we observed all CS-MGIT-resistant isolates harbored mutations in the *alr* gene, including three previously known high-confidence mutations M343T, T20M, and L113R, as well as the novel R243S mutation. We also found that mutations could lead to CS resistance by disrupting protein stability.

## INTRODUCTION

Tuberculosis (TB) is a notifiable infectious disease caused by *Mycobacterium tuberculosis* complex (MTBC). TB and drug-resistant tuberculosis (DR-TB) (multidrug-resistant, MDR/rifampicin-resistant, RR-TB) remain a major threat to global public health. An estimated 10.8 million persons worldwide developed TB, and 400,000 of those cases resulted in MDR/RR-TB in 2023 (1). The effective management of DR-TB relies upon accurate diagnosis and treatment. To design effective regimens against MDR/RR-TB, it is essential to provide comprehensive and precise predictions of antibiotic resistance.

Cycloserine (CS) is a widely used second-line drug for DR-TB treatment. CS interferes with peptidoglycan formation and bacterial cell wall synthesis by inhibiting the activity of two enzymes, alanine racemase and d-alanine ligase (2–4). Studies revealed that mutations in the *ald* and *alr* genes confer CS resistance in MTBC (5–7). In 2018, the World Health Organization (WHO) recommended the use of CS as one of the group B drugs for DR-TB treatment. Since culture-based phenotypic drug susceptibility testing (DST) is still considered the gold standard for drug resistance detection, the WHO has recently set a critical concentration (CC) of 16 µg/mL for CS-MGIT DST (8).

To improve the management of DR-TB, Taiwan has implemented a designated, government-organized, and hospital-initiated program, the Taiwan MDR-TB Consortium (TMTC), providing comprehensive and high-quality medical care for MDR-TB cases since 2007 (9). Clinical physicians adhere to the most up-to-date international guidelines for TB diagnosis and treatment. To strengthen our TB laboratory program, we performed CS resistance analyses to provide comprehensive DST. We combined genotypic DST (gDST) and phenotypic DST (pDST) results to analyze CS resistance in DR-TB cases in Taiwan and suggest a CC for CS. In addition, we explored the molecular features of isolates harboring mutations to elucidate and understand the impact of specific mutations on protein stability and functions in relation to drug resistance.

## MATERIALS AND METHODS

### Study design and isolates

This retrospective study included initial MTBC isolates from 114 MDR/RR-TB cases, including six rifampin-phenotypically sensitive isolates with *rpoB* disputed mutations. Universal DST for culture-positive MTBC isolates was implemented in Taiwan. Using the gDST results as the reference, we evaluated the performances of pDST methods, the broth microdilution (BMD) method, and Bactec MGIT 960 system (MGIT) across various critical concentrations or breakpoints. We proposed interim critical concentrations based on the highest concordance between gDST and pDST results. Information on characteristics and outcomes of studied cases was obtained from the National TB Registry.

This study was approved by the Institutional Review Board of Centers for Disease Control, Ministry of Health and Welfare (TwCDC IRB No. 109205 and No. 110108), and analyzed only archived MTBC, and thus, written informed consent from the participants was waived. Cultivation and processing of MTBC were performed in a certified biosafety level 3 laboratory. All methods were performed in accordance with the relevant guidelines and regulations.

### Phenotypic drug susceptibility testing

MTBC isolates were subjected to DST using the agar proportion method (APM) with 7H10 and 7H11 media (Becton, Dickinson and Company, Spark, MD, USA). Drug resistance was defined as the growth of 1% of colonies in a drug-containing medium. According to the WHO recommendations, the critical concentrations of the tested drugs in the 7H10 medium were as follows: rifampicin (RIF), 1 µg/mL; isoniazid (INH), 0.2 and 1 µg/mL; ethambutol (EMB), 5 and 10 µg/mL; streptomycin (SM), 2 and 10 µg/mL; moxifloxacin (MFX), 0.5 µg/mL; and levofloxacin (LFX), 1 µg/mL (10). The critical concentrations of the tested drugs in the 7H11 medium were as follows: rifabutin (RFB), 0.5 µg/mL; kanamycin (KM), 6 µg/mL; amikacin (AMK), 6 µg/mL; capreomycin (CM), 10 µg/mL; ethionamide (ETO), 10 µg/mL; and para-aminosalicylic acid (PAS), 8.0 µg/mL (10, 11). The resistance to pyrazinamide (PZA, 100 µg/mL) and bedaquiline (BDQ, 1 µg/mL), clofazimine (CFZ, 1 mg/mL), linezolid (LZD, 1 mg/mL), and delamanid (DLM, 0.06 mg/mL) was tested using MGIT, as described previously (10). The growth on the control medium was compared to that on the drug-containing medium to determine the susceptibility. The DST results were categorized as resistant or susceptible, and the tests were validated by determining the susceptibility of *Mycobacterium tuberculosis* H37Rv, used as a quality control strain. RR-TB is defined as an MTBC isolate resistant to RIF. MDR-TB is defined as an MTBC isolate resistant to at least RIF and INH. Pre-XDR-TB is defined as an RR/MDR-TB isolate resistant to any fluoroquinolones (FQs). XDR-TB is defined as an RR/MDR-TB isolate resistant to any FQs and at least one additional Group A drug (12).

Phenotypic-MIC testing for MTBC isolates was performed using the Bactec MGIT 960 system (Becton, Dickinson and Company, Spark, MD, USA) and the Sensititre *Mycobacterium tuberculosis* MYCOTB plates (Thermo Scientific, TREK Diagnostic Systems, UK). In the MGIT system, CS concentrations tested were 4, 8, 16, 32, and 64 µg/mL, and the growth on drug-containing media was compared to that on control media to determine the susceptibility and MICs. The MYCOTB plates are 96-well microtiter plates containing 12 drugs (RIF, INH, EMB, SM, RFB, ofloxacin [OFX], MFX, KM, AMK, ETO, PAS, and CS). The MICs were determined following the manufacturer’s instructions. The H37Rv strain was used as the control in each lot of testing, and the results were interpreted by two independent readers.

### Genotypic drug susceptibility testing

For drug resistance genetic assessment, we used published information and pDST results for the interpretation of mutations in the *al* gene conferring resistance to CS (13).

### Sanger sequencing

One loop (0.5 µL) of bacteria was placed into a microtube and resuspended in 500 µL of Tris-EDTA buffer. The bacterial liquid was inactivated at 95°C for 20 min. The bacterial lysate was centrifuged at 12,000 × *g* for 1 min, and the supernatant was used as a template for PCR. In this study, we analyzed two CS resistance-associated genes, namely, *ald* and *alr*. The specific primers were designed based on *Mycobacterium tuberculosis* strain H37Rv (GenBank: AL123456.3) to amplify the whole genes by PCR (Table 1). PCRs were performed using a HotStarTaq Master Mix kit (QIAGEN, Germany). Each reaction mixture contained 12.5 µL of 2 × HotStarTaq Master Mix (QIAGEN, Germany), 0.5 µL of each primer (10 µM), and 5 µL of the bacterial lysate. Double-distilled water was added to the mixture to obtain a total volume of 25 µL. The PCR conditions were as follows: initial denaturation at 95°C for 10 min; 35 cycles of 95°C for 1 min, 56°C–62°C (according to the optimal primer annealing temperature) for 1 min, and 72°C for 1 min; and a final elongation step of 72°C for 6 min. The PCR products were analyzed using the capillary electrophoresis QIAxcel Advanced system (QIAGEN, Germany). The DNA sequence was confirmed by Sanger sequencing (Genomics BioSci & Tech, Taiwan). In addition, sequence assembly and mutation identification were performed using Sequencher (Gene Codes Corporation, USA) software.

**TABLE 1:** PCR primer, Tm, and amplicon size of CS resistance-associated genes

Primer	Sequence (5' to 3')	Tm (°C)	Amplicon size (bp)
*ald*-1F	GCAAGCTCAGGATTCTGCGGTCC	58	818
*ald*-1R	GCGTCGAGTTGCCGAAGTTTGTCG		
*ald*-2F	GGCGCTTACCACCTGATGCGAACC	56	851
*ald*-2R	CACCAGATGACGAAGCCGATACGG		
*alr*-1F	CGCTCCCGTCAATTCATGCCGG	62	805
*alr*-1R	GGAATCGTCAGGCTTGTCGGCG		
*alr*-2F	CCATGCTGACCGCGTTACGCC	58	803
*alr*-2R	CCAGCGTTAGGGTGTCCTCGACG		

### Whole-genome sequencing and bioinformatics analysis

Genomic DNA was extracted following the phenol-chloroform method and quantified using a Qubit 4.0 fluorometer (Thermo Fisher Scientific, Waltham, MA, USA). Paired-end libraries were prepared using a TruSeq DNA PCR-free high-throughput (HT) sample preparation kit (Illumina, Inc., San Diego, CA, USA) according to the manufacturer’s protocol. The average fragment size (500 to 600 bp) of the DNA libraries was estimated by an Agilent 2100 Bioanalyzer. The 24 purified DNA libraries were pooled, and the DNA concentration was quantified with a Qubit 4.0 fluorometer. The pooled libraries (11 pM) were sequenced on an Illumina MiSeq system (Illumina, Inc.) with a MiSeq reagent kit version 3 (600 cycles), which showed that the first paired-end reads were 350 nucleotides (nt) in length, whereas the second paired-end reads were 250 nt in length. Sequencing reads were checked using fastQC (https://www.bioinformatics.babraham.ac.uk/projects/fastqc/) as a primary assessment of data quality and then analyzed using the TB-Profiler tool for drug resistance prediction (14).

### Predicting the effects of mutations

We used mCSM (15), DynaMut2 (16), and DDMut (17) to calculate Gibbs free energy (ΔΔG) changes to predict the impact of point mutations on protein stability (ΔΔG <0, stable mutations; ΔΔG >0, unstable mutations). The effects of mutations near the front end of a gene or protein, as well as certain types of mutations except for single-point mutations, cannot be predicted and are interpreted as NA.

### Statistical analyses

The sensitivity, specificity, and concordance were calculated for each test method at each critical concentration/tested. The agreement between phenotypic and genotypic DST results was evaluated by kappa and 95% confidence interval (CI) values. The kappa result was interpreted as follows: values of 0.81 to 1.00 indicated almost perfect agreement, values of 0.61 to 0.80 indicated substantial agreement, values of 0.41 to 0.60 indicated moderate agreement, values of 0.21 to 0.40 indicated fair agreement, and values of <0.20 indicated slight to no agreement (18). The chi-squared test or Fisher’s exact test (when expected cell size <5) was used for univariate analysis of categorical variables. A value of *P* < 0.05 was considered to indicate statistical significance. Odds ratios (ORs) and 95% confidence intervals were calculated to estimate the correlation between the CS MIC and variables. To determine the relationship between MICs measured by MGIT and Sensititre, we performed a Pearson correlation analysis (19). Descriptive parameters, including slope (a), intercept (b), and coefficient of determination (R²), were calculated.

## RESULTS

### Characteristics of the study population

Of the 114 study cases, 27 (23.7%), 71 (62.3%), 9 (7.9%), 1 (0.9%), and 6 (5.3%) isolates were RR-TB, MDR-TB, pre-XDR-TB, XDR-TB and other drug-resistant cases, respectively (Table 2). In addition, 82 (71.9%) were male, the median age was 61.6 years (IQR: 45.9–75.7) and 97 (85.1%) were new cases. Chest radiography revealed cavitation in 23 (20.2%) cases, whereas 95 (83.3%) and 9 (7.9%) cases had pulmonary TB and pleural effusion, respectively. We found 58 (50.9%) and 79 (69.3%) cases were AFB smear-positive and infected with lineage 2 isolates, respectively.

**TABLE 2:** Demographic and clinical characteristics of 114 tuberculosis cases^*a*^

Characteristics	No. of cases (%)	No. (%) of isolates		Univariate analysis	
		CS MGIT MIC ≥ 32 µg/mL	CS MGIT MIC ≤ 16 µg/mL	Odds ratio (95% CI)	*P*-value^*b*^
	114 (100)	5 (4.4)	109 (95.6)		
Gender					
Male	82 (71.9)	5 (6.1)	77 (93.9)	NA	0.320^#^
Female	32 (28.1)	0 (0.0)	32 (100.0)	Ref.	
Age					
≤40	20 (17.5)	0 (0.0)	20 (100.0)	NA	0.585^#^
41–60	32 (28.1)	2 (6.3)	30 (93.8)	1.756 (0.279–11.031)	0.619^#^
≥61	62 (54.4)	3 (4.8)	59 (95.2)	1.271 (0.204–7.912)	1.000^#^
Case category					
New	97 (85.1)	4 (4.1)	93 (95.9)	0.688 (0.072–6.560)	1.000^#^
Previously treated	17 (14.9)	1 (5.9)	16 (94.1)	Ref.	
AFB smear					
Positive	58 (50.9)	2 (3.4)	56 (96.6)	0.631 (0.101–3.926)	0.676^#^
Negative	48 (42.1)	2 (4.2)	46 (95.8)	0.913 (0.147–5.687)	1.000^#^
Scanty	8 (7.0)	1 (12.5)	7 (87.5)	3.643 (0.358–37.117)	0.310^#^
Chest radiography					
Normal	4 (3.5)	0 (0.0)	4 (100.0)	NA	1.000^#^
Abnormal with cavitation	23 (20.2)	1 (4.3)	22 (95.7)	0.989 (0.105–9,293)	1.000^#^
Abnormal without cavitation	86 (75.4)	4 (4.7)	82 (95.3)	1.317 (0.141–12.299)	1.000^#^
Abnormal but not related to TB	1 (0.9)	0 (0.0)	1 (100.0)	NA	1.000^#^
Site of tuberculosis					
Pulmonary	95 (83.3)	5 (5.3)	90 (94.7)	NA	0.588^#^
Extrapulmonary	19 (16.7)	0 (0.0)	19 (100.0)	Ref.	
Pleural effusion					
Yes	9 (7.9)	0 (0.0)	9 (100.0)	NA	1.000^#^
No	105 (92.1)	5 (4.8)	100 (95.2)	Ref.	
Lineage					
Lineage 1	7 (6.1)	0 (0.0)	7 (100.0)	NA	1.000^#^
Lineage 2	79 (69.3)	5 (6.3)	74 (93.7)	NA	0.184^#^
Lineage 4	26 (22.8)	0 (0.0)	26 (100.0)	NA	0.344^#^
Bovis	2 (1.8)	0 (0.0)	2 (100.0)	NA	1.000^#^
Drug resistance profile					
RR	27 (23.7)	0 (0.0)	27 (100.0)	NA	0.337^#^
MDR	71 (62.3)	4 (5.6)	67 (94.4)	2.508 (0.271–23.204)	0.648^#^
Pre-XDR	9 (7.9)	1 (11.1)	8 (88.9)	3.156 (0.314–31.688)	0.342^#^
XDR	1 (0.9)	0 (0.0)	1 (100.0)	NA	1.000^#^
Other	6 (5.3)	0 (0.0)	6 (100.0)	NA	1.000^#^
Drug resistance					
to rifampicin	108 (94.7)	5 (4.6)	103 (95.4)	NA	1.000^#^
to isoniazid	83 (72.8)	5 (6.0)	78 (94.0)	NA	0.321^#^
to ethambutol	53 (46.5)	5 (9.4)	48 (90.6)	NA	**0.020^#^**
to pyrazinamide	33 (28.9)	2 (6.1)	31 (93.9)	1.677 (0.267–10.530)	0.626^#^
to levofloxacin	9 (7.9)	1 (11.1)	8 (88.9)	3.156 (0.314–31.688)	0.342^#^
to moxifloxacin	10 (8.8)	1 (10.0)	9 (90.0)	2.778 (0.280–27.570)	0.374^#^
to streptomycin	42 (36.8)	5 (11.9)	37 (88.1)	NA	**0.006^#^**
to amikacin	4 (3.5)	0 (0.0)	4 (100.0)	NA	1.000^#^
to capreomycin	5 (4.4)	0 (0.0)	5 (100.0)	NA	1.000^#^
to kanamycin	7 (6.1)	0 (0.0)	7 (100.0)	NA	1.000^#^
to rifabutin	93 (81.6)	5 (5.4)	88 (94.6)	NA	0.582^#^
to ethionamide	27 (23.7)	4 (14.8)	23 (85.2)	14.957 (1.594–140.369)	**0.011^#^**
to bedaquiline	6 (5.3)	0 (0.0)	6 (100.0)	NA	1.000^#^
to clofazimine	4 (3.5)	0 (0.0)	4 (100.0)	NA	1.000^#^
to linezolid	0 (0.0)	0 (0.0)	0 (0.0)	NA	1.000^#^
to delamanid	1 (0.9)	0 (0.0)	1 (100.0)	NA	1.000^#^
to para-aminosalicylic acid	8 (7.0)	2 (25.0)	6 (75.0)	11.444 (1.597–82.023)	**0.039^#^**

^*a*^ AFB smear, acid-fast bacilli smear; RR, rifampicin-resistant; MDR, multidrug-resistant; Pre-XDR, pre-extensively drug-resistant; XDR, extensively drug-resistant; OR, odds ratio; CI, confidence interval; Ref., reference; and NA, not applicable due to a small no. of cases.

^*b*^ #Fisher's exact probability test (two-tailed).

Based on the CC suggested by the WHO, we analyzed the characteristics and clinical outcomes of CS-resistant isolates with MGIT MIC ≥ 32 µg/mL (Table 2). Of the 5 (4.4%) CS-resistant cases, 4 and 1 were MDR-TB and Pre-XDR-TB, respectively. We found that CS resistance was not associated with gender, age, case category, AFB smear, clinical manifestations, or genotypes. Nevertheless, a significant association between isolates with higher CS-MGIT MIC values and concurrent resistance to EMB, SM, ETO, or PAS was observed (*P* < 0.05).

### Phenotypic drug susceptibility testing

#### MGIT MIC

**Fig 1:**
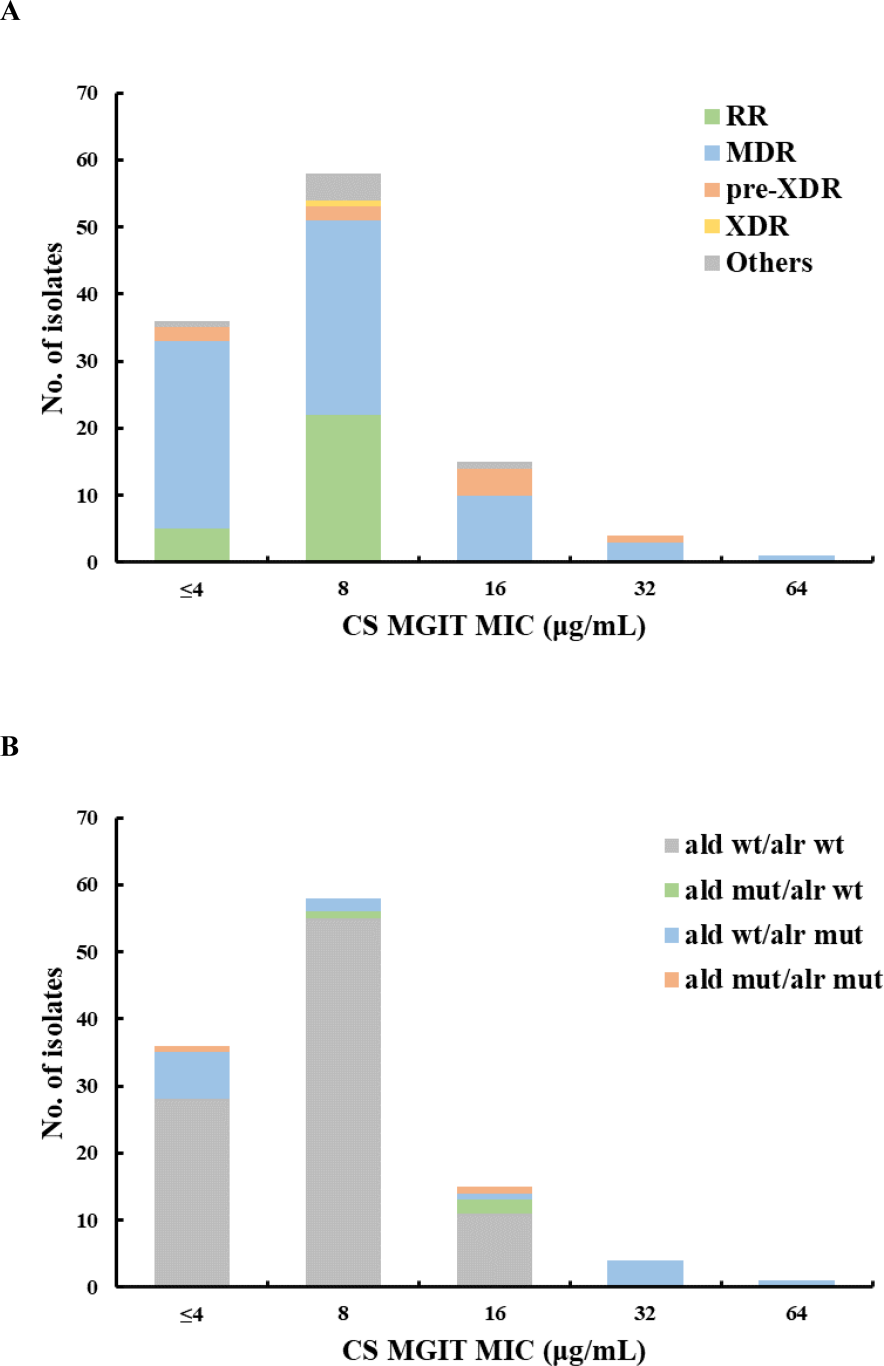
Distribution of cycloserine (CS) MGIT MIC values stratified to drug resistance profiles (**A**) or gene mutations (**B**) among 114 MTBC isolates. Rifampicin-resistant (RR), multidrug-resistant (MDR), pre-extensively drug-resistant (pre-XDR), extensively drug-resistant (XDR) isolates, and others. WT, wild-type; Mut, mutant.

The CS-MGIT MIC values of the 114 isolates ranged from ≤4 to 64 µg/mL. The MIC_50_, MIC_90_, and MIC_99_ values were 8, 16, and 32 µg/mL, respectively. We observed a unimodal CS-MGIT MIC distribution that peaked at 8 µg/mL (58/114, 50.9%) (Fig. 1). The reference H37Rv strain had an MIC of 16 µg/mL, which was consistent with previously published studies (20, 21).

Of the 114 isolates, 20 isolates (17.5%) harbored mutations, and 4 (3.5%) carried silent mutations in the *ald* or *alr* gene (Fig. 1B). We analyzed the performance of CS-MGIT at each CC or breakpoint tested. The optimal consistency with gDST was achieved with the CC of 16 µg/mL, which aligns with that suggested by the WHO. The concordance was 93.0%, and the corresponding Kappa values were 0.526 (95% CI 0.246–0.805); the sensitivity and specificity were 38.5% and 100.0%, respectively (Table 3).

**TABLE 3:** The performance of DST for different pDST methods at each critical concentration or breakpoint tested^*a*^

Method (No. of isolates)	CC/BP (μg/mL)	pDST	gDST		Performance			
			R	S	Sensitivity (%)	Specificity (%)	Concordance (%)	Kappa (95% CI)
MGIT (114)	32	R	1	0	7.7	100.0	89.5	0.129 (0.000–0.358)
		S	12	101				
	16	R	5	0	38.5	100.0	93.0	0.526 (0.246–0.805)
		S	8	101				
	8	R	9	11	69.2	89.1	86.8	0.473 (0.248–0.697)
		S	4	90				
Sensititre (113^*b*^)	64	R	1	0	7.7	100.0	89.4	0.129 (0.000–0.357)
		S	12	100				
	32	R	1	1	7.7	99.0	88.5	0.106 (0.000–0.329)
		S	12	99				
	16	R	3	1	23.1	99.0	90.3	0.316 (0.027–0.604)
		S	10	99				
	8	R	6	1	46.2	99.0	92.9	0.565 (0.300–0.830)
		S	7	99				

^*a*^ CC, critical concentration; BP, breakpoint; pDST, phenotypic drug susceptibility testing; gDST, genotypic drug susceptibility testing; R, resistant; S, susceptible; CI, confidence interval.

^*b*^ The MIC result for one isolate cannot be determined.

#### Sensititre MIC

**Fig 2:**
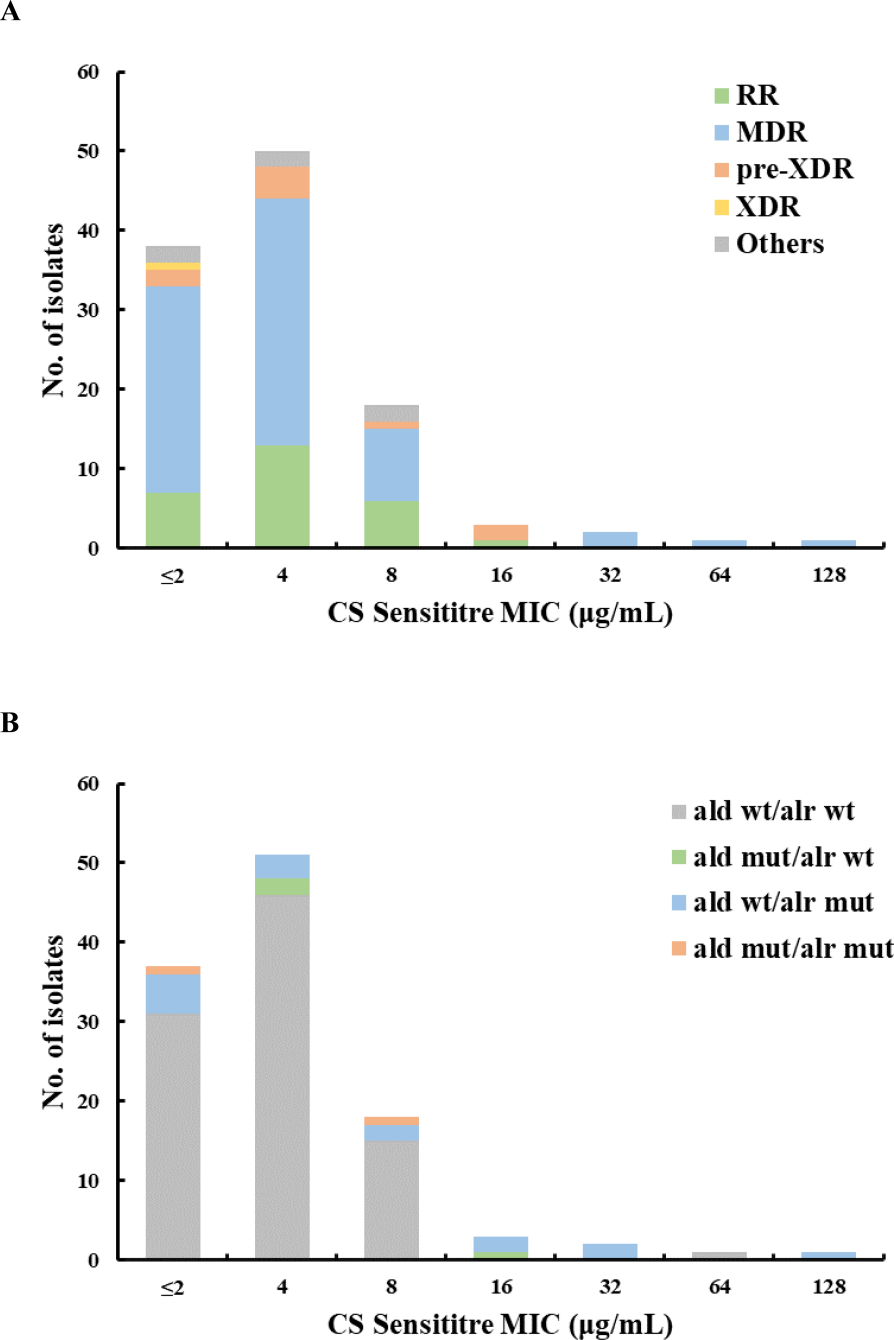
Distribution of cycloserine (CS) Sensititre MICs value stratified to drug resistance profiles (**A**) or gene mutations (**B**) among 113 MTBC isolates. Rifampicin-resistant (RR), multidrug-resistant (MDR), pre-extensively drug-resistant (pre-XDR), extensively drug-resistant (XDR) isolates, and others. WT, wild-type; Mut, mutant.

The CS-Sensititre MIC values of the 113 isolates ranged from ≤2 to 128 µg/mL. The MIC_50_, MIC_90_, and MIC_99_ values were 4, 8, and 64 µg/mL, respectively (Fig. 2). We observed a unimodal CS-Sensititre MIC distribution. The peak of the distribution was at 4 µg/mL. (50/113, 44.2%) (Fig. 2). The reference H37Rv strain had an MIC of 4–8 μg/mL, which was consistent with a previously published study (20). We analyzed the performance of CS-Sensititre at each CC or breakpoint tested. The optimal consistency with gDST was achieved with the CC of 8 µg/mL. The concordance was 92.9%, and the corresponding Kappa values were 0.565 (95% CI 0.300–0.830). The sensitivity and specificity were 46.2% and 99.0%, respectively (Table 3). Furthermore, we analyzed the correlation of MICs determined by MGIT and Sensititre, finding a correlation coefficient (r) of 0.729 (Fig. S1). This suggests a significant relationship between the two methodologies, supporting their use in parallel to assess drug susceptibility.

#### Genotypic DST

Of the 20 isolates harboring mutations, 15 (75.0%) isolates harbored mutations in the *alr* gene including five novel mutations, M1G (t-71g, *N* = 1), G17R (g-24a, *N* = 1), Q30R (Q6R, *N* = 7), R243S (R219S, *N* = 1), and S261N (S237N, *N* = 1); and three known mutations, T20M (c-14t, *N* = 1), L113R (L89R, *N* = 1), and M343T (M319T, *N* = 2). We found three (15.0%) isolates carried *ald* mutations, including three novel mutations, M1I (*N* = 1), E118K (*N* = 1), and A184T (*N* = 1). In addition, two (10.0%) isolates harbored a novel double mutation, *ald* 266_a_deletion and *alr* R403C (Table 4). We observed five CS-MGIT-resistant isolates harbored mutations in the *alr* gene, including previously known mutations M343T (*N* = 2), T20M (*N* = 1), L113R (*N* = 1), and a novel mutation R243S (*N* = 1). Using the pDST results as references, we verified that the aforementioned known mutations were high-confidence CS-resistance (6, 22). Besides, the MTB genome sequencing project entry for *alr* (GenBank accession no.: Z77165) indicates a GTG codon at the 5′ end of the *alr* open reading frame as the start codon for alanine racemase. However, Strych et al. suggested an ATG codon 72 nucleotides further downstream is more likely to be the start codon (23). Therefore, we presented two different *alr* mutation scenarios, as shown in Table 4.

**TABLE 4:** The characteristics of isolates with mutations and the effect on protein stability^*a*^^,^^*e*^

Gene (Mutation)		No. of isolates	CS MIC (μg/mL)		Predicted stability change (ΔΔG, kcal/mol)^*d*^					
					Alanine dehydrogenase			Alanine racemase		
Ald	Alr		MGIT	Sensititre	mCSM	DynaMut2	DDMut	mCSM	DynaMut2	DDMut
M1I^*b*^	WT	1	16	4	**−0.90**	**−0.21**	**−7.86**	–	–	–
E118K^*b*^	WT	1	16	4	**−0.36**	**−0.25**	**−0.84**	–	–	–
A184T^*b*^	WT	1	8	16	**−1.46**	**−1.3**	**−1.85**	–	–	–
nt 266 a del (Fs)^*b*^	R403C (R379C)^*b*^	2	≤4, 16	≤2, 8	NA	NA	NA	**−0.25**	0.79	0.13
WT	M1G (t-71g)^*b*^	1	≤4	4	–	–	–	NA	NA	NA
WT	G17R (g-24a)^*b*^	1	8	16	–	–	–	NA	NA	NA
WT	T20M (c-14t)^*c*^	1	32	16	–	–	–	NA	NA	NA
WT	Q30R (Q6R)^*b*^	7	≤4, ≤4, ≤4, ≤4, ≤4, ≤4, and 8	≤2, ≤2, ≤2, ≤2, ≤2, 4, and 4	–	–	–	NA	NA	NA
WT	L113R (L89R)^*c*^	1	32	32	–	–	–	**−0.96**	**−0.57**	**−0.55**
WT	R243S (R219S)^*b*^	1	32	8	–	–	–	**−1.62**	**−1.39**	**−0.95**
WT	S261N (S237N)^*b*^	1	16	8	–	–	–	**−1.42**	**−0.94**	**−0.32**
WT	M343T (M319T)^*c*^	2	32 and 64	32 and 128	–	–	–	**−2.14**	**−1.45**	**−2.65**

^*a*^ WT, wild-type; Fs, frameshift mutation; NA, not available.

^*b*^ Novel mutation.

^*c*^ Previously reported mutation.

^*d*^ The free energy (ΔΔG) was calculated for point mutations in the available protein structures by using mCSM, DynaMut2, DDMut. ΔΔG < 0, and stabilizing mutations; ΔΔG > 0, destabilizing mutations.

^*e*^ "–", not applicable, the mutation does not occure in the corresponding gene bold terms mean "stablizing".

We used WGS data to explore known mutations in genes presumably related to CS resistance including *ddlA*, *cycA*, *Rv3331*, *Rv3772*, and *Rv1435c*. However, we only identified some mutations like lineage markers, including *ddla* T365A, *cycA* R93L, *Rv3331* P423L, *Rv3772* T142A, and *Rv1435c* t-85c.

### Predicting the effects of single-point mutations

The *ald* and *alr* genes encode alanine dehydrogenase (ALD) and alanine racemase (ALR), respectively. Table 4 showed predictions of the effect of mutations on protein stability. We found mutations M1I, E118K, and A184T in the *ald* gene and L113R, R243S, S261N, and M343T in the *alr* gene, which were predicted to have destabilizing effects. Notably, the *alr* R403C showed discordant results from three prediction methods. The effect was expected to be stable when analyzed with DDMut and DynaMut2, while destabilized with mCSM.

## DISCUSSIONS

The WHO recommends CS as a drug for DR-TB treatment and recently suggested a CC of 16 µg/mL for CS-MGIT DST (8, 24). In this study, we found five (4.4%) isolates were resistant to CS, all of which harbored mutations in the *alr* gene, including one isolate with the novel R243S mutation. A study revealed that CS resistance rates were 4.6% and 20.0% in MDR-TB and XDR-TB cases, respectively (21). Besides, we reassured that M343T, T20M, and L113R in the *alr* gene are associated with CS resistance (6, 22), whereas the novel Q30R is not.

The characteristics and clinical outcomes of CS-resistant and CS-susceptible isolates are shown in Table 2. We found no association between characteristics/genotypes and CS resistance, whereas there was a notable correlation between higher CS MIC values and resistance to other drugs like EMB, SM, ETO, or PAS. This suggests that CS resistance is associated with multidrug resistance. Wu et al. further revealed that the proportion of CS resistance is notably higher in XDR-TB cases compared to MDR-TB cases, highlighting the complexity of managing drug resistance (21).

We found the optimal consistency was achieved with the CC of 16 µg/mL for CS-MGIT DST, which aligned with the CC suggested by the WHO and prior studies (8, 21, 25). This indicates that 16 µg/mL is an optimal threshold for assessing CS resistance using MGIT. However, the CC was lower than the 64 µg/mL proposed by Deshpande et al. (20). Cross-study parallel comparisons can be difficult. Various MIC values suggested in different studies could be due to the adopted research methodology or composition of the cohort. In addition, a previous study revealed that CS continuously degraded in the culture medium, and the degradation was sufficient to alter the MIC value (26).

CS resistance is primarily associated with mutations in the *ald* and *alr* genes. In this study, we found 15 (75.0%), 3 (15.0%), and 2 (10.0%) isolates had *alr*, *ald,* and *alr*/*ald* double mutations, respectively. All CS-resistant isolates harbored mutations in the *alr* gene but not in the *ald* gene, which was consistent with observations of previous studies (21, 27). Wu et al. identified just two *ald* mutations among seven isolates with mutations conferring CS resistance, while Chen et al. found mutations only in the *alr* gene, but not in the *ald* gene. Since mutations in the *ald* gene are rare, all novel *ald* variants identified in this study need further verification.

Notably, we found that the *alr* R403C showed discordant results from three protein stability prediction tools in this study. With DDMut and DynaMut2, it was predicted to be stable, whereas it was unstable using mCSM. Unlike mCSM, which typically assumes a static protein structure and overlooks the implications of mutations within its conformational landscape, DynaMut2 integrates normal mode analysis with a graph-based representation of the protein structure (16). Furthermore, DDMut predicts changes using deep learning models, utilizing both forward and hypothetical reverse mutations to accommodate model antisymmetry (17). Therefore, DDMut and DynaMut2 tools might lead to more accurate predictions in the impact of missense mutations on protein stability. However, we observed distinct CS MICs in two isolates with the *alr* R403C mutation. Due to the limited number of strains with the *alr* R403C mutation, further studies are required to elucidate its impact on protein stability and its role in conferring CS resistance. Furthermore, we assessed the impact of point mutations on protein stability based on ΔΔG; and stable mutations were characterized by ΔΔG <0, while unstable mutations had ΔΔG >0. However, we acknowledge that the concept of “stability” could involve different aspects, such as protein degradation or folding efficiency. Further experimental validation studies, such as thermal stability assays, protein degradation studies, or structural analysis, would be needed to comprehensively demonstrate the instability caused by these mutations.

This study had limitations. First, Taiwan had low drug-resistant prevalence and second-line DST was only performed mainly for DR-TB cases. This study analyzed relatively few samples, and the exclusion of drug-susceptible isolates might introduce the possibility of sampling bias, which may impact the final conclusions. Nevertheless, we have already provided our data set to the WHO CS-DST working group for more comprehensive analysis. Another studied isolates with mutations in the *ald* or *alr* genes were rare, and analysis of the association with CS-resistance or treatment outcome was not feasible. Second, the CC ranges were 4 to 64 µg/mL and 2 to 128 µg/mL for CS-MGIT and CS-Sensititre, respectively. In this study, only one isolate had an MIC of 64 µg/mL, and the narrow range of CC distribution may not be sufficient to accurately estimate the tentative epidemiological cut-off (TECOFF) value.

In conclusion, we confirm that the CC for CS-MGIT is reliable to determine CS resistance. In addition, an MIC of 8 µg/mL could be considered an interim CC in CS-Sensititre. We verified that the variants *alr* M343T, T20M, and L113R were correlated with CS resistance, whereas the novel mutation *alr* Q30R was not. Although the proportion of CS-resistant strains is relatively low, we found that isolates resistant to EMB, SM, ETO, or PAS tended to exist at higher CS MIC values. To achieve better treatment outcomes, a comprehensive DST should be conducted before prescribing CS for the treatment of DR-TB.

## SUPPLEMENTAL MATERIAL

Figure S1 spectrum.03422-24-s0001.pdf Correlation of cycloserine (CS) MICs value determined by MGIT and Sensititre. The MGIT MIC and Sensititre MIC were plotted for each MTBC isolate. The dotted line represents a regression line.

ASM does not own the copyrights to Supplemental Material that may be linked to, or accessed through, an article. The authors have granted ASM a non-exclusive, world-wide license to publish the Supplemental Material files. Please contact the corresponding author directly for reuse.
