# Supplementary material for: Cycloserine resistance among drug-resistant tuberculosis cases in Taiwan
Source: Microbiol Spectr. 2025 Jun 9;13(7):e03422-24. doi: 10.1128/spectrum.03422-24 (PMC12211041; doi:10.1128/spectrum.03422-24)
Supplement: Figure S1 — Correlation of cycloserine (CS) MICs value determined by MGIT and Sensititre. The MGIT MIC and Sensititre MIC were plotted for each MTBC isolate. The dotted line represents a regression line. [file spectrum.03422-24-s0001.pdf]

**Supplementary Figure**

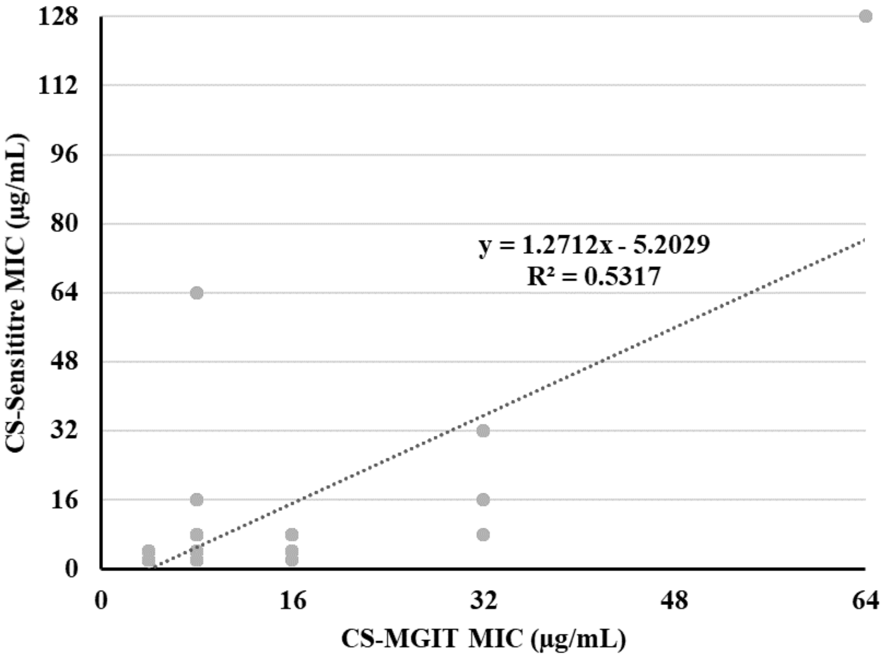


**FIG S1.** Correlation of cycloserine (CS) MICs value determined by MGIT and Sensititre. The MGIT MIC and Sensititre MIC were plotted for each MTBC isolate. The dotted line represents a regression line.
